# Role of non-coding RNAs and RNA modifiers in cancer therapy resistance

**DOI:** 10.1186/s12943-020-01171-z

**Published:** 2020-03-02

**Authors:** Xinyi Zhang, Kai Xie, Honghua Zhou, Yuwei Wu, Chan Li, Yating Liu, Zhaoya Liu, Qian Xu, Shuang Liu, Desheng Xiao, Yongguang Tao

**Affiliations:** 1 Key Laboratory of Carcinogenesis and Cancer Invasion, Ministry of Education, Department of Pathology, Xiangya Hospital, School of Basic Medicine, Central South University, Changsha, 410078 Hunan China; 2 Department of Cardiovascular Medicine, Xiangya Hospital, Central South University, Changsha, 410008 Hunan China; 3 Department of Cardiovascular Medicine, Third Xiangya Hospital, Central South University, Changsha, 410013 Hunan China; 4 Department of Neurosurgery, Xiangya Hospital, Central South University, Changsha, 410008 Hunan China; 5 Department of Geriatrics, Third Xiangya Hospital, Central South University, Changsha, 410013 Hunan China; 6 Department of Cardiovascular Surgery, Xiangya Hospital, Central South University, Changsha, 410008 Hunan China; 7 Department of Oncology, Institute of Medical Sciences, National Clinical Research Center for Geriatric Disorders, Xiangya Hospital, Central South University, Changsha, 410008 Hunan China; 8 NHC Key Laboratory of Carcinogenesis (Central South University), Cancer Research Institute, Central South University, Changsha, 410078 Hunan China; 9 Hunan Key Laboratory of Early Diagnosis and Precision Therapy, Department of Thoracic Surgery, Second Xiangya Hospital, Central South University, Changsha, 410011 China

**Keywords:** ncRNAs, RNA modifiers, Chemoresistance, Radioresistance

## Abstract

As the standard treatments for cancer, chemotherapy and radiotherapy have been widely applied to clinical practice worldwide. However, the resistance to cancer therapies is a major challenge in clinics and scientific research, resulting in tumor recurrence and metastasis. The mechanisms of therapy resistance are complicated and result from multiple factors. Among them, non-coding RNAs (ncRNAs), along with their modifiers, have been investigated to play key roles in regulating tumor development and mediating therapy resistance within various cancers, such as hepatocellular carcinoma, breast cancer, lung cancer, gastric cancer, etc. In this review, we attempt to elucidate the mechanisms underlying ncRNA/modifier-modulated resistance to chemotherapy and radiotherapy, providing some therapeutic potential points for future cancer treatment.

## Introduction

Cancer is a life-threaten disease worldwide and produces a financial burden to society. With the advance of technologies, some treating approaches, like chemotherapy and radiotherapy, have been gradually developed and turned out to be effective within patients. Among them, chemotherapy is the first-line standard treatment plan for most cancer patients, especially when they are not appropriate for surgical treatment. There are approximately hundreds of drugs approved for cancer, with more in development, and the mechanisms beyond them are diverse, mainly focusing on elements that suppress fundamental function and kill proliferating cells. These drugs include compounds with pleiotropic effects such as DNA-modifying agents (cisplatin/DDP), drugs that involve in the key physiological pathways like metabolic activities (methotrexate/MTX), and molecules targeting hormone such as estrogen receptor mediators (tamoxifen/TAM) [1]. Besides, radiotherapy is another adjuvant method and has been already employed to treat at least half of cancer patients worldwide. The principle of treatment is based on the theory that tumor cells have limited ability to repair damaged DNA and tend to divide faster, thus cancerous areas are more sensitive to radiation than normal tissues, while the normal tissue parts surrounding the tumor can withstand radiation therapy and recover [2]. Although both chemotherapy and radiotherapy have extended the disease-free and/or overall survival among patients, an unavoidable issue has slowly emerged that tumor cells tend to be resistant to chemicals or radiation, indicating the existence of therapy resistance mechanisms in tumor cells. Both intrinsic and acquired resistance significantly limit the effectiveness of therapies, resulting in poor prognosis, tumor metastasis and recurrence [3]. Addressing this issue is an urgent challenge nowadays.

Non-coding RNAs (ncRNAs), accounting for 98% of the human genome, are classified into several types considering the length, which include long non-coding RNAs (lncRNAs), microRNAs (miRNAs), circular RNA (circRNAs), other types like small nuclear RNAs (snRNAs), small nucleolar RNAs (snoRNAs), piwi-interacting RNAs (piRNAs), small interfering RNAs (siRNAs) and so on. LncRNAs are a kind of ncRNAs that are greater than 200 nucleotides, with a high abundance, various modes of action, and mainly involved in regulation of cellular physiological and pathological activities. According to the ENCODE project, it is estimated that the human genome encodes more than 28,000 different lncRNAs [4]. Comprehensive analysis of their expression in multiple human organs and brain regions displays that lncRNAs are generally lower expressed than protein-coding genes, and show more tissue-specific expression patterns [5]. The role of lncRNAs is investigated by interaction with macromolecules of cells. Chromatin-binding lncRNAs can regulate gene expression by changing local chromatin structure or recruiting regulatory molecules to specific sites. Interaction of lncRNAs with multiple proteins can promote the assembly of protein complexes or weaken the interaction between proteins. Interaction between mRNAs and lncRNAs can affect mRNAs stability or translation or isolation. Recent studies have even found that lncRNAs can serve as a precursor of miRNA or circRNA [6]. Besides, miRNAs are a family of small ncRNAs, around 22 nucleotides, which play an important role in biological pathways among multicellular organisms including mammals. MiRNAs can exert their effects by silencing multiple mRNAs and regulating the expression of various oncogenes or tumor suppressor genes post-transcriptionally [7]. It has been documented that miRNAs involve in various cancers, including breast, colon, gastric, lung, prostate and ovarian. In some cases, miRNAs can interact with lncRNAs and then form the network to regulate tumorigenesis [8]. Additionally, circRNAs were previously thought to be erroneous splicing processes that had no function. However, the increasing amount of extensive investigations indicate that circRNAs can act as gene regulators, or even can be coded into proteins [9, 10]. Nowadays, numerous researches have been conducted to determine the relationship between cancer and circRNAs, suggesting circRNAs can be treated as the biomarkers in cancer. Furthermore, other types, such as snRNAs, snoRNAs, piRNAs and siRNAs, have also gained considerable attention owing to their substantial tumor-regulated role in numerous cancers. Among them, snRNAs are a collection of small nuclear RNAs, around 150 nucleotides, and proved to be stable, abundant, universal, highly conserved and nuclear-localized. Each snRNA, like U1, U2—naming from the high uridine (U) content, normally combines with specific proteins to form small nuclear ribonucleoproteins (snRNPs), mainly involving in the processing of pre-messenger RNA (hnRNA) in the nucleus [11]. Similarly, snoRNAs are a class of small RNA molecules nearly 60–300 nucleotides and can bind to snRNPs to form small nucleolar ribonucleoproteins (snoRNPs) complexes. The biological function of snoRNAs is to guide chemical modifications of other RNAs, including ribosomal RNAs, transfer RNAs and small nuclear RNAs. From a structural basis, snoRNAs fall into two categories termed box C/D snoRNAs (SNORDs) for methylation and box H/ACA snoRNAs (SNORAs) for pseudouridylation [12]. Others like piRNAs, with a length of 30 nucleotides, are recognized as the largest class of small ncRNAs. PiRNAs commonly form RNA-protein complexes through PIWI protein family to play the regulatory role, like silencing the transcriptional gene process, maintaining germline and stem cell functions, regulating translation and mRNA stability [13]. And about siRNAs, a kind of double-stranded ncRNA molecules with a length of about 21 to 25 nucleotides, can combine with the target mRNA through a completely complementary pairing. This interferes with the expression of specific genes by degrading mRNA after transcription, preventing translation [14]. Besides, the modification of ncRNAs, mediating by RNA modifiers, is also an aspect that should not be ignored when understanding the mechanism of RNA action. RNA modifications are very common and diverse in cells, which may produce changes in structure and stability, thereby achieving the regulation of cellular physiological and pathological activities [15, 16].

In this review, we focus exclusively on the ncRNAs and their modifiers mediated pro-tumorigenic/anti-tumorigenic response to different cancer therapies. LncRNAs, miRNAs, circRNAs, and other types are discussed, highlighting the major contribution of lncRNAs and miRNAs to chemotherapy and radiotherapy resistance. Investigating the potential mechanisms will help understand tumorigenesis and metastasis, providing some new and fresh insights to develop novel drugs and drug combinations, biomarker application and translational medicine to improve anticancer therapy efficiency.

### LncRNAs and cancer therapy resistance

In recent years, there has been an explosion of research focused on investigating the role of multiple lncRNAs in regulating recurrence and metastasis of malignancies, including proliferation, migration, expansion, immortality, angiogenesis, et al. [17, 18]. Especially, the relationship between lncRNAs and cancer therapy resistance has received increased attention due to the complicated interaction network. This section highlights the function of lncRNAs in different cancers, mainly on modulating therapy resistance.

### LncRNAs in cancer chemoresistance

**Fig. 1:**
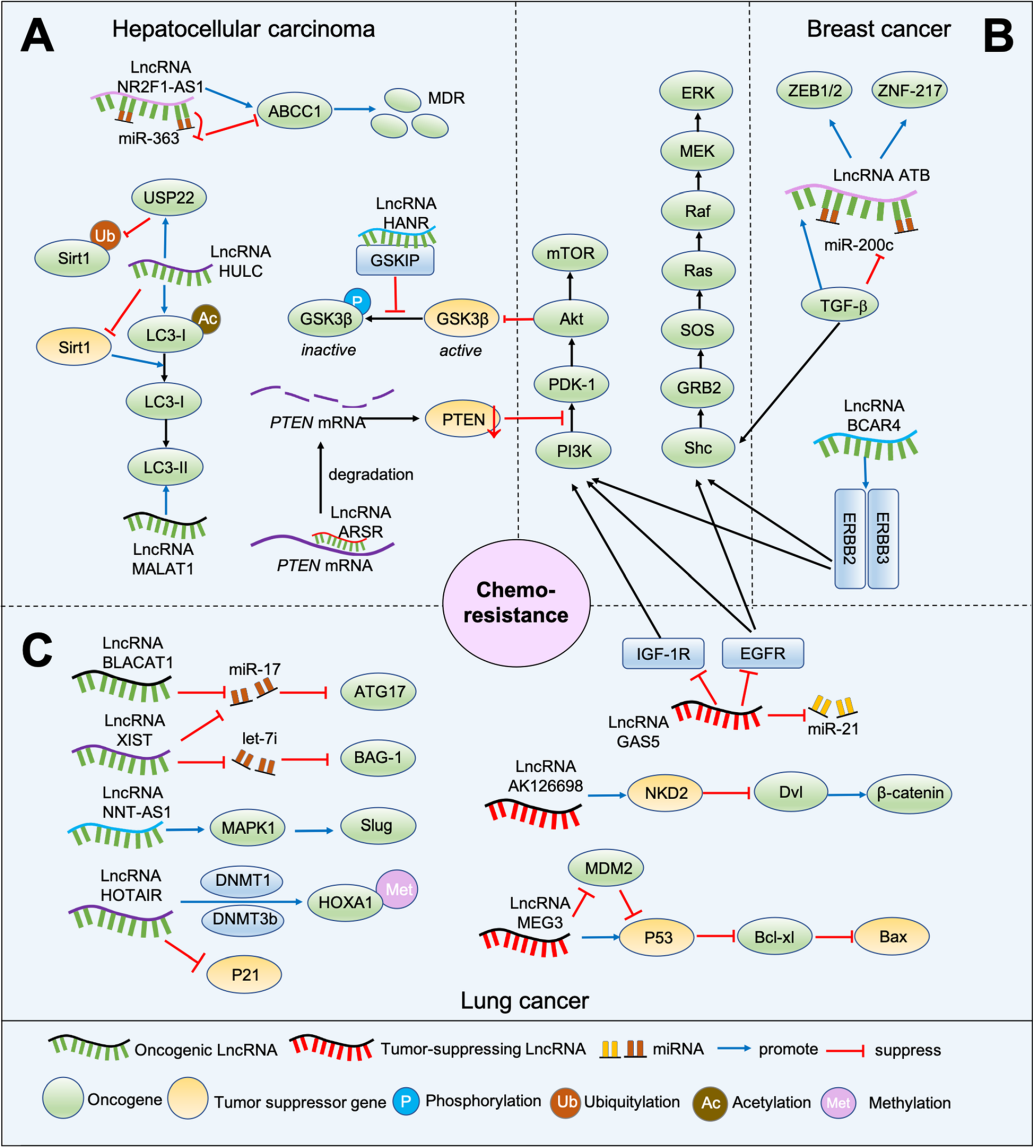
Overview of the involvement of lncRNAs in cancer chemoresistance. **a** Different lncRNAs-related pathways in HCC. **b** Different lncRNAs-related pathways in BC. **c** Different lncRNAs-related pathways in lung cancers

As essential molecules on regulating tumor progression, lncRNAs have been considered as mediators involving in different mechanisms of chemoresistance, such as altering drug efflux, interfering DNA damage repair, triggering apoptosis, inducing mutations of drug targets, etc. [19]. Moreover, most of the lncRNAs have been revealed to promote chemoresistance, while very few have the inhibitory influence. The lncRNAs involved in cancer chemoresistance and their modulated pathways are illustrated in Fig. 1.

#### Hepatocellular carcinoma (HCC)

Worldwide, liver cancer ranks the second most lethal tumor and among all cases, HCC alone accounts for 90% [20]. Patients with advanced HCC mostly benefit from chemotherapy with doxorubicin (DOX), sorafenib, 5-fluorouracil (5-FU) and platinum drugs. Unfortunately, due to the acquisition of multi-drug resistance (MDR), some patients are likely to develop a poor response to chemotherapy, resulting in metastasis, recurrence and bad prognosis [21]. Multiple lncRNAs have been discovered to involve in this part (Fig. 1a). For DOX resistance, HCC associated long non-coding RNA (HANR), which is dramatically up-regulated in HCC tissues, can antagonize the sensitivity of HCC to DOX and lead to poor prognosis via GSK3β interaction protein (GSKIP)/P-GSK3β pathway. HANR directly binds to GSKIP and diminishes the phosphorylation of GSK3β, mediating glycogen metabolism and cell growth, which reduces the chemotherapy sensitivity. Conversely, silencing the expression of HANR limits HCC proliferation in vitro and in vivo, as well as inducing apoptosis and promoting sensitivity to DOX [22]. Besides, lncRNA activated in renal cell carcinoma with sunitinib resistance (lncARSR), which is also overexpressed in HCC, has been identified to take part in DOX resistance through phosphatase and tensin homolog-phosphatidylinositol 3-kinase (PTEN-PI3K)/Akt pathway. LncARSR firstly links with *PTEN* mRNA to promote its degradation, then decreases PTEN expression and stimulates the PI3K/Akt signaling pathway, which finally results in drug resistance in vitro and in vivo [23]. Except for DOX, some other lncRNAs have been documented to be correlated with platinum drug resistance, like oxaliplatin. LncRNA NR2F1-AS1 is aberrantly expressed in oxaliplatin-resistant HCC tissues and can enhance tumor invasion in vitro and in vivo. This phenomenon depends on the induction of miR-363/ATP-binding cassette subfamily c member 1 (ABCC1) signaling. LncRNA NR2F1-AS1 can trigger ABCC1 expression through miR-363, and as a member of the superfamily of ATP-binding cassette (ABC) transporters, ABCC1 is usually associated with MDR, therefore linking NR2F1-AS1 with oxaliplatin resistance [24]. Additionally, some lncRNAs are responsible for multiple drug resistance. For instance, highly upregulated lncRNA in HCC (lncRNA HULC) suppresses the chemosensitivity of oxaliplatin, 5-FU and pirarubicin (THP) in HCC by inducing autophagy through inhibiting silent information regulator 1(Sirt1) protein. It can also increase the expression of ubiquitin-specific peptidase 22(USP22) and decrease the ubiquitin-mediated degradation of Sirt1 protein by cutting the conjugated polyubiquitin chain of Sirt1 [25]. Similarly, metastasis-associated lung adenocarcinoma transcript 1 (MALAT1) has been investigated to be robustly upregulated in 5-FU, DOX, and mitomycin resistant HCC. The silencing of MALAT1 can reverse drug resistance, reduce LC3-II level and enhance 5-FU-induced apoptosis [26]. Taken together, it is plausible that various lncRNAs contribute to different mechanisms of drug resistance in HCC via modulating downstream pathways, like inhibiting phosphorylation, involving in metabolism, inducing MDR expression, and so on, which provides some new ideas for improvement of therapies.

#### Breast cancer (BC)

BC is the most common noncutaneous malignancy among women in the world [27]. Based on gene expression profile, BC has been classified into various subtypes, including luminal A and B, basal-like, human epidermal growth factor receptor 2 (HER2) enriched and normal breast-like subgroups [28]. Among them, for patients with estrogen receptor (ER)-positive, estrogen stimulation and antihormone therapy, like estrogen and TAM, is the most common treatment in clinics. However, there are always some unpredictable scenarios to patients and they even develop serious chemoresistance. Some lncRNAs have been discovered to involve in TAM resistance (Fig. 1b). LncRNA HOX antisense intergenic RNA(HOTAIR), which is found to be remarkably upregulated in TAM-resistant BC, can enhance the disease progression and aggravation, indicating HOTAIR can be used as a potential therapeutic target in TAM-resistant patients [29]. Besides, breast cancer antiestrogen resistance 4 (BCAR4) also contributes to TAM-resistance. As a powerful oncogene, it antagonizes the sensitivity of BC to estrogen stimulation and antihormone therapy, partially through the ERBB2/ERBB3 pathway. Therefore, patients with an increased level of BCAR4 are easily to be resistant to chemotherapy [30, 31]. Similarly, colon cancer-associated transcript 2 (CCAT2) can enhance proliferation and reduce apoptosis in TAM-resistant cells, and depletion of CCAT2 provides a new approach for patients [32]. Except for TAM, lncRNAs are also noted to be associated with trastuzumab resistance (Fig. 1b). For example, lncRNA activated by TGF-β (lnc-ATB), overexpressed in trastuzumab-resistant BC, can trigger drug resistance, promote metastasis and result in poor prognosis via competitively sponging miR-200c, then activating zinc finger E-box binding homeobox 1 (ZEB1) and ZNF-217, ultimately inducing epithelial-mesenchymal transition (EMT) [33]. Thus, these findings provide novel thoughts into the role of lncRNAs play on BC drug resistance, most of them contributing to tumor aggressiveness and dissemination by promoting downstream pathways, and it is necessary to identify more lncRNAs that can be potential therapeutic targets for chemoresistant BC patients.

#### Lung cancer

The platinum-based chemotherapy is the major treatment for lung cancer patients. Unfortunately, large amounts of patients have been reported to develop MDR [34]. In DDP-resistant cells, several lncRNAs have been differentially expressed (Fig. 1c). HOTAIR, for example, is easy to induce drug resistance and by the siRNA-mediated silencing method, the sensitivity can partially rescue. P21 is revealed to be the downstream of HOTAIR and an increased level of p21 functionally reverse the HOTAIR-induced DDP resistance in vitro. What’s more, HOTAIR can recruit homeobox A1 (HOXA1) by RNA immunoprecipitation. The silencing of HOTAIR decreases HOXA1 methylation and then increases the chemotherapy in small cell lung cancer (SCLC) [35]. In contrast, tumor suppressor lncRNA maternally expressed gene 3 (MEG3) expression is reduced in DDP-resistant cells. Exogenous MEG3 can mediate the expression of p53 and Bcl-xl, then restoring the DDP resistance in vitro [36]. Moreover, lincAK126698 has been shown to interfere with DDP-induced apoptosis through inhibiting naked cuticle homolog 2 expression and enhancing β-catenin expression [37]. Similarly, lncRNA-XIST can reduce DDP efficacy in tumor cells via the lncRNA-XIST/miR-17/autophagy regulatory axis and the let-7i/BAG-1 axis by decreased apoptosis and increased proliferation [38, 39]. Moreover, it has been reported that lncRNA bladder cancer-associated transcript 1 (BLACAT1) is upregulated in DDP-resistant NSCLC cells and promotes autophagy and chemoresistance via the miR-17/autophagy-related 7 (ATG7) signaling pathway [40]. LncRNA nicotinamide nucleotide transhydrogenase-antisense RNA1 (lncRNA NNT-AS1) is remarkably expressed in DDP-resistant NSCLC tissues and cells, and overexpression of lncRNA NNT-AS1 can alter cell proliferation, cell cycle and apoptosis through the mitogen-activated protein kinase (MAPK)/Slug signaling pathway [41]. LncRNAs also contribute to gefitinib efficacy (Fig. 1c). Upregulation of growth arrest special 5 (GAS5) has turned out to shrink tumor growth by its key downstream mediator insulin-like growth factor 1 receptor (IGF-1R) [42]. Conversely, inhibition of linc00635–001 during gefitinib therapy can downregulate Akt expression and reverse drug resistance [43]. Therefore, dysfunction of various lncRNAs in lung cancer can enhance chemoresistance or chemosensitivity in different ways, mainly focusing on apoptosis and autophagy.

#### Gastric cancer (GC)

As the fourth most common malignancy and the second leading cause of death in the world, resistance to therapies for GC is a big challenge and needs more effort to overcome. For example, in DDP-resistant GC cell lines and tissues, lncRNA plasmacytoma variant translocation 1 (PVT1) is robustly expressed and shown the anti-apoptotic characteristic by upregulating MDR1, multidrug resistance-associated protein 1 (MRP1), mammalian target of rapamycin (mTOR) and hypoxia-inducible factor (HIF)-1α, which facilitates the development of chemoresistance [44]. Similarly, overexpression of lncRNA AK022798 enhances the level of MRP1, as well as reducing apoptotic-related genes, like *caspase 3* and *caspase 8*, and then antagonizes the sensitivity of GC to DDP [45]. Conversely, some lncRNAs have been proved to sensitize GC to 5-FU. As a tumor-suppressing lncRNA, a specifically downregulated lncRNA in GC (LEIGC) can functionally inhibit EMT, tumor growth and cell proliferation, resulting in the improvement of chemosensitivity in GC [46]. Collectively, these studies emphasize that lncRNAs involve in various regulations of GC to drug resistance, like proliferation, apoptosis and EMT, providing more evidence that lncRNAs can be used as predictors on the prognosis of chemotherapy in GC.

#### Other cancers

Chemotherapy is proved to be the standard first-line treatment for various cancers, while the therapy-resistance mediating by lncRNAs usually results in poor prognosis. In bladder cancer, for example, lncRNAs can inhibit apoptosis and promote proliferation. Urothelial cancer-associated 1 (UCA1) has been found to be overexpressed in bladder cancer cell lines and induce less apoptosis after DDP treatment, partially through activating the wingless-type MMTV integration site family member 6 (Wnt6) signaling pathway [47, 48]. In pancreatic cancer, knockdown of lncRNA HOXA transcript at the distal tip (HOTTIP) enhances the chemosensitivity to gemcitabine via consistently modulating HOXA13 level, and overexpression of the HOTTIP/HOXA13 axis indicates poor prognosis and aggressive stage [49]. In osteosarcoma (OS), PVT1 contributes to gemcitabine resistance by activating the c-MET/PI3K/Akt pathway, which is largely relied on miR-152, resulting in enhancement of cell proliferation and reduction of apoptosis [50]. As a result, lncRNA-induced chemoresistance has been widely found in multiple cancers and is mechanically modulated through different pathways, such as proliferation and apoptosis, and more investigations are needed for further application.

### LncRNAs in cancer radioresistance

**Fig. 2:**
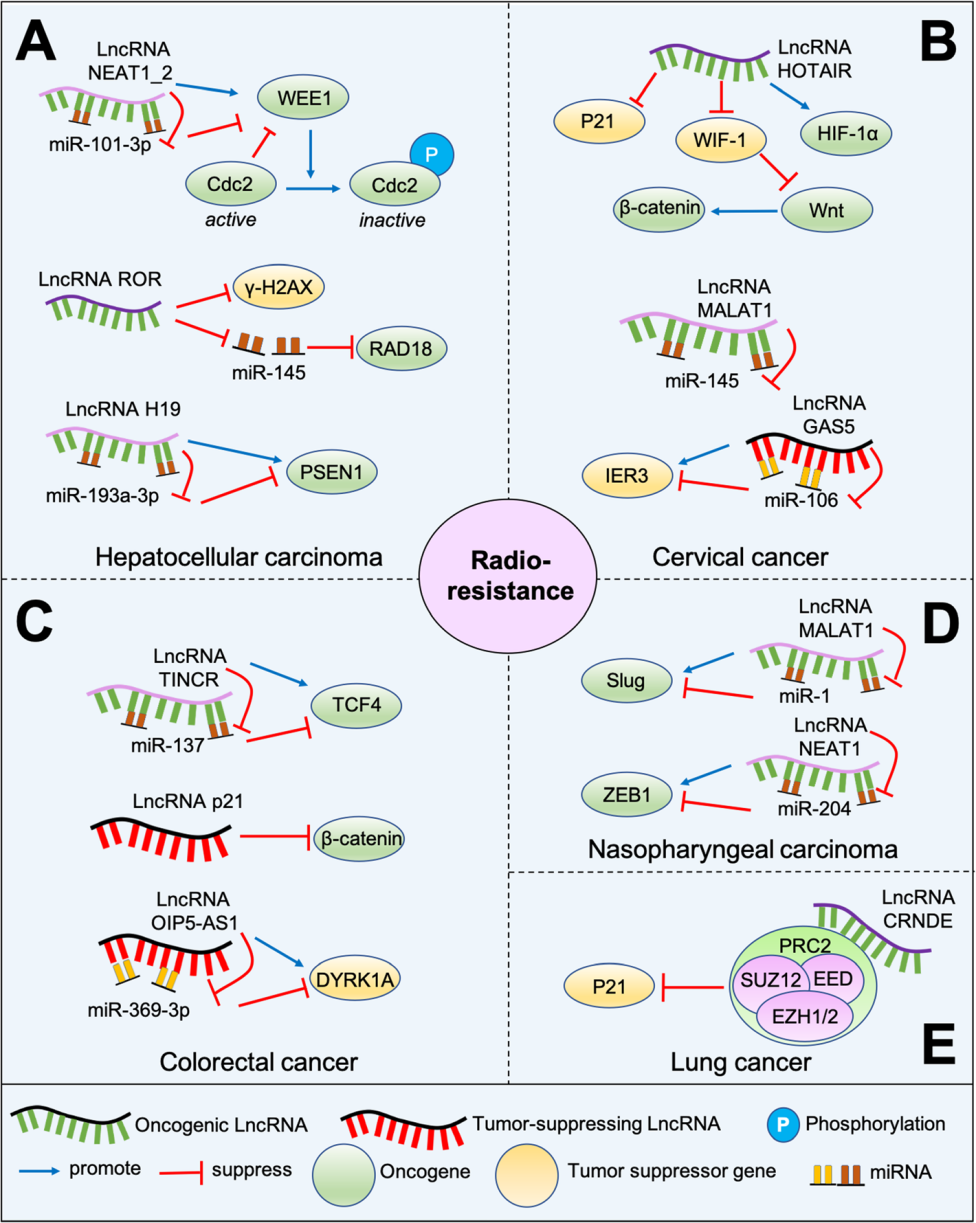
Overview of the involvement of lncRNAs in cancer radioresistance. **a** Different lncRNAs-related pathways in HCC. **b** Different lncRNAs-related pathways in CC. **c** Different lncRNAs-related pathways in CRC. **d** Different lncRNAs-related pathways in NPC. **e** Different lncRNAs-related pathways in lung cancer

Radiation therapy has grown up to be an essential tool in the treatment of cancer patients. However, due to biological complexities and heterogeneities, as well as the presence of cancer stem cells (CSCs) [51, 52], certain types of tumors are proved to be more resistant to radiation therapy, resulting in tumor recurrence, metastasis and poor prognosis [53]. Studies have shown that lncRNAs can modulate radioresistance through multiple aspects, including DNA damage repair, apoptosis, EMT control, and tumor stem cell activity. The lncRNAs involved in cancer radioresistance and their modulated pathways are illustrated in Fig. 2.

#### HCC

Radiotherapy is another choice for patients with HCC. It has been observed that lncRNAs involve in the regulation of DNA repair and then reduce radioresistance (Fig. 2a). Normally, damaged DNA is repaired during G1 arrest; while due to deficiency of G1-S checkpoint, cancer cells usually rely on G2-M checkpoint to fix DNA damage. For example, WEE1 kinase, one type of tyrosine kinases, facilitates DNA repair by arresting G2-M cell cycle checkpoint before entering mitosis [54]. The lncRNA NEAT1_2/miR-101-3p/WEE1 axis has been demonstrated to be related to radiotherapy resistance in HCC. Upregulation of lncRNA NEAT1_2 and WEE1, along with miR-101-3p downregulation, can promote the proliferation of cancer cells under radiation conditions and then decrease therapy sensitivity [55]. Similarly, linc-ROR, known as long non-coding RNA regulator of reprogramming, is overexpressed in radioresistant HCC cell lines. Linc-ROR knockdown can increase therapy efficiency in vitro and in vivo via inhibiting DNA repair capacity. Moreover, it can mediate RAD18 expression by working as a competing endogenous RNA (ceRNA) for miR-145, resulting in enhancement of DNA repair and radioresistance [56]. Besides, lnc TP73-AS1, which is highly expressed in radioresistant tumor tissues, has been reported to induce radiation resistance in liver cancer through the PTEN/Akt signaling pathway. The silencing of lnc TP73-AS1 can decrease cell proliferation, increase apoptosis and therefore enhance radiosensitivity [57]. Also, another pathway, the lncRNA H19/miR-193a-3p axis, has been found to induce therapeutic tolerance by targeting presenilin 1 (PSEN1), a key component of γ secretase, and then promote tumor aggravation [58]. Overall, these studies suggest that lncRNAs contribute to radioresistance in HCC via multiple ways, mainly resulting from dysfunction of DNA damage repair, which can be used as a meaningful aspect for further research and even in the clinical trial.

#### Cervical cancer (CC)

CC is one of the most common gynecological malignancies in the world [59]. Its morbidity and mortality have been increasing year by year. So far, patients with CC are widely treated with the combination of surgery, radiotherapy or chemotherapy. However, due to the therapy resistance, they are easy to get poor prognosis, with a five-year survival rate of only 40 to 50% [60]. Some lncRNAs have been found to involve in radioresistance (Fig. 2b). HOTAIR can interact with the Wnt signaling, and then promote autophagy, enhance EMT, increase cell proliferation and suppress apoptosis after radiotherapy [61]. HOTAIR can also inhibit p21 [62] and increase HIF-1α expression in irradiated CC cells to trigger radioresistance [63]. Besides, the regulation of cell metabolism also plays an important role in the production of radiotherapy resistance. Lnc UCA1, along with the enzyme hexokinase-2 (HK2), has been found to enhance glycolysis in radioresistant tumor cells, thus participating in the regulation of radiotherapy sensitivity [64]. In addition, MALAT1 is able to reduce the G2-M phase arrest rate and inhibit apoptosis of irradiated tumor cells by sponging miR-145 [65]. Different from the above lncRNAs, the overexpression of lncRNA GAS5 has been discovered to enhance the radiotherapy sensitivity of tumor cells. It is responsible for upregulating immediate early response 3 (IER3) expression through inhibiting miR-106b. Therefore, the axis of GAS5/IER3/miR-106b can promote the sensitivity of tumor cells to radiation [66]. Taken together, these results demonstrate that various lncRNAs take part in the efficiency of radiation and most of them induce radioresistance via promoting proliferation, mediating metabolism, and arresting cell cycle.

#### Nasopharyngeal carcinoma (NPC)

Various mechanisms have been found to contribute to the radiotherapy resistance in NPC (Fig. 2d). For example, MALAT1 can mediate the activity of CSCs and trigger radioresistance by interacting with the miR-1/slug axis [67]. LncRNA NEAT1 has been documented to promote the expression of ZEB1 by sponging miR-204, so as to play a role in regulating EMT and tumor resistance to radiotherapy [68]. Besides, lnc PVT1 has been proved to be overexpressed in NPC cells. The silencing of PVT1 induces radiosensitivity by inhibiting proliferation and enhancing apoptosis, which indicates that PVT1 can be used as a potential therapeutic target [69]. Thus, lncRNAs involve in the process of CSCs regulation, apoptosis and EMT, leading to the radioresistance in NPC.

#### Colorectal cancer (CRC)

CRC is the leading cause of death in malignancies. Patients with CRC commonly get effective radiotherapy; while some of them develop radioresistance, which results in poor prognosis [70]. It has been found that the role of lncRNAs in radiotherapy resistance in CRC includes two aspects: apoptosis and CSCs (Fig. 2c). For instance, overexpression of terminal differentiation-induced non-coding RNA (TINCR) is proved to induce radioresistance in CRC cell lines. The silencing of TINCR inhibits transcription factor 4 (TCF4) by mediating miR-137 expression, and TCF4 is related to CSCs function [71]. In contrast, lncRNA-p21 has been discovered to decrease β-catenin signaling transduction, and therefore weaken the viability, self-renewal, and glycolysis of CSCs in vitro, which finally promotes the radiosensitivity of CRC [72]. Similarly, lncRNA OIP5-AS1 can improve the radiosensitivity of tumor cells via inhibiting the expression of miR-369-3p, thus increasing the expression of dual-specificity tyrosine-(Y)-phosphorylation regulated kinase 1A (DYRK1A), the downstream gene of miR-369-3p. Overexpression of lncRNA OIP5-AS1, as well as DYRK1A, facilitates apoptosis of tumor cells after irradiation [73]. What’s more, lncRNA UCA1 is upregulated in CRC and induces therapy resistance. Suppression of UCA1 decreases colony formation, proliferation, and EMT, at the same time, triggers apoptosis and G2-M arrest [74]. In addition, HOTAIR is also related to the regulation of apoptosis after radiotherapy, showing that it can be used as a potential therapeutic target [75]. Overall, these studies prove that the apoptosis and CSCs-like properties mediated by lncRNAs may be the crucial part to overcome the radioresistance in CRC patients.

#### Other cancers

LncRNAs have been also found to be related to radiotherapy resistance in other cancers. As a ceRNA, lncRNAs can regulate the expression of downstream genes by acting on miRNA. For example, in laryngeal cancer, DiGeorge syndrome critical region gene 5 (DGCR5) can sponge miR-195 [76] and miR-506 [77], to induce CSCs-like characteristics in radioresistant tumor cells. In bladder cancer, it has been proved that the silencing of lncRNA Taurine-upregulated gene 1 (TUG1) improves radiation sensitivity by inhibiting HMGB1 expression, resulting in less cell proliferation, more cell apoptosis and decreased colony survival in bladder cancer cell lines under radiation [78]. LncRNAs can even directly interact with proteins. In lung cancer, the lncRNA colorectal neoplasia differentially expressed (CRNDE) can bind with polycomb-repressive complex 2 (PRC2) and then recruit its key part EZH2 to p21 (cyclin-dependent kinase inhibitor 1A/CDKN1A) promoter regions and inhibit its transcription. As a result, this CRNDE/PRC2/CDKN1A axis mechanistically contributes to radioresistance in lung cancer cells via modifying the G1-S transition and triggering apoptosis [79] (Fig. 2e). Besides, lncRNAs have also been reported to change the cell cycle through other pathways. In prostate cancer (PC), knockdown of UCA1 changes Akt signaling and arrests cell cycle in the G2-M transition period, thus enhancing radiosensitivity, decreasing proliferative capacity and disrupting cell cycle progression [80]. As a result, lncRNAs contribute to radiation efficacy in multiple cancers, most of them related to radioresistance mainly by inducing CSCs-like properties, increasing proliferation and altering cell cycle, while some of them linked with radiosensitivity via promoting apoptosis, indicating the therapeutic potential of lncRNAs in future cancer treatment.

### MiRNAs and cancer therapy resistance

MiRNAs play crucial roles in the regulation of target oncogenes and tumor suppressor genes at the posttranscriptional level. Aberrant miRNAs expression is commonly implicated in the development and progression of tumor cells, as well as therapy resistance [81, 82]. In this part, we systematically summarize the literatures on miRNAs modulating chemoresistance and radioresistance, providing some fresh insight into miRNAs-induced cancer therapy resistance.

### MiRNAs in cancer chemoresistance

Different miRNAs show different roles in terms of chemoresistance or chemosensitivity, and some miRNAs exhibit double-edged effects. The miRNAs involved in cancer chemoresistance and their modulated targets/pathways are listed in Table 1.

**Table 1:** Cancer chemoresistance related miRNAs

Cancers	miRNAs	Drugs	Targets/Mechanisms	References
Breast cancer	miR-5195-3p	Paclitaxel	Targeting EIF4A2	[83]
	miR-542-3p	Paclitaxel	Suppressing survivin	[84]
	miR-181c	Doxorubicin	Inactivating *OPN*; Enhancing p-53-dependent transactivation and apoptosis	[85]
	miR-381	Doxorubicin	Downregulating MAPK/FYN signaling	[86]
		Cisplatin	Targeting *MDR1*	[87]
	miR-100	Cisplatin	Suppressing HAX-1; Modulating mitochondrial apoptosis pathway	[88]
Hepatocellular carcinoma	miR-19a-3p	Sorafenib	Modulating PTEN/Akt pathway	[89]
	miR-142-3p	Sorafenib	Targeting ATG5 and ATG16L1	[90]
	miR-3129-5p	Doxorubicin	Enforcing mRNA stability of ZEB1	[91]
	miR-760	Doxorubicin	Increasing PTEN expression; Decreasing the phosphorylation of Akt	[92]
	miR-21-5p	Cisplatin	Downregulating FASLG	[93]
	miR-16	Paclitaxel	Targeting IKBKB/NF-kB signaling pathway	[94]
Lung cancer	miR-219a-5p	Cisplatin	Negatively regulating FGF9	[95]
	miR-539	Cisplatin	Targeting on DCLK1	[96]
	miR-181b	Cisplatin	Inactivating Notch2/Hes1 pathway	[97]
	miR-9	Cisplatin	Negatively modulating EIF5A2	[98]
	miR-133b	Cisplatin	Inhibiting GSTP1	[99]
	miR-130b	Cisplatin	Targeting PTEN	[100]
	miR-935	Paclitaxel	Regulating SOX7	[101]
Colorectal cancer	miR-128-3p	Oxaliplatin	Negatively modulating Bmi1 and MRP5	[102]
	miR-483-3p	Oxaliplatin	Increasing FAM171B	[103]
	miR-135b	Oxaliplatin	Regulating FOXO1/Bim/Noxa axis	[104]
	miR-195-5p	5-FU	Suppressing Notch2/RBPJ signaling; Inhibiting GDPD5	[105, 106]
	miR-148a	Cisplatin	Modulating WNT10b and β-catenin pathway	[107]
Ovarian cancer	miR-142-5p	Cisplatin	Targeting multiple anti-apoptotic genes including *XIAP, BIRC3, BCL2, BCL2L2, MCL1*	[108]
	miR-378a-3p	Cisplatin	Targeting MAPK1/GRB2	[109]
	miR-363	Cisplatin	Modulating snail-induced EMT	[110]
	miR-139-5p	Cisplatin	Inactivation of MAPK pathway and RNF2	[111]
	miR-139	Cisplatin	Inhibition of ATP7A/B	[112]
	miR-34a	Cisplatin	Blocking HDAC1	[113]
	miR-330-5p	Cisplatin	Downregulating S100A7	[114]
	miR-149-5p	Cisplatin	Targeting the core kinase components of the Hippo signaling pathway, MST1, SAV1; Inhibiting TEAD transcription	[115]
	miR-514	Cisplatin	Interacting with ATP binding cassette subfamily	[116]
	miR-129	Paclitaxel	Regulating UCA1/miR-129/ABCB1 axis	[117]
	miR-874	Paclitaxel	Regulating miR-874/serine/ SIK2 axis	[118]
	miR-383-5p	Paclitaxel	Regulating miR-383-5p/TRIM27 axis	[119]
	miR-1246	Paclitaxel	Regulating miR-1246/Cav1/p-gp/M2-type macrophage axis	[120]
Glioblastoma	miR-151a	Temozolomide	Suppressing XRCC4-mediated DNA repair	[121]
	miR-519a	Temozolomide	Targeting STAT3/Bcl-2/Beclin-1 pathway	[122]
	miR-224-3p	Temozolomide	Modulating HIF-1α/miR-224-3p/ATG5 axis	[123]
	miR-1238	Temozolomide	Regulating CAV1/EGFR pathway	[124]
	miR-501-3p	Cisplatin	Targeting MYCN	[125]
Gastric carcinoma	miR-494	Doxorubicin	Targeting the 3’UTR region of PDE4D	[126]
	miR-6785-5p/ miR-642a-3p	5-FU	Regulating FOXO4	[127]
	miR-17	Cisplatin, 5-FU	Modulating DEDD	[128]
	miR-193a-3p	Cisplatin	Regulating mitochondrial apoptosis pathway	[129]
	miR-155-5p	Paclitaxel	Modulating GATA3 and TP53INP1	[130]
Pancreatic carcinoma	miR-200b	Gemcitabine	Reversing EMT	[131]
	miR-125a-3p	Gemcitabine	Reversing EMT	[132]
	miR-301	Gemcitabine	Regulating cadherin 1 and inducing EMT	[131]
Osteosarcoma	miR-340	Cisplatin	Targeting ZEB1	[133]
	miR-233	Cisplatin	Forming miR-233/Hsp70/JNK/JUN/ miR-233 feedback loop	[134]
Papillary thyroid carcinoma	miR-206	Euthyrox	Blocking p38 and JNK signaling pathway via targeting MAP 4 K3	[135]

#### BC

Except for lncRNAs mentioned before, miRNAs are also proved to get involved in the resistance to different chemotherapies. As one of the standard treatments, paclitaxel (PTX) is widely used in triple-negative BC (TNBC) and HER2-overexpressed BC. It has been shown that miR-5195-3p is downregulated in PTX-resistant tumor tissues and cell lines, while the upregulation of miR-5195-3p can increase drug sensitivity via targeting eukaryotic translation initiation factor 4A2 (EIF4A2) [83]. Similarly, miR-542-3p mimic exhibits not only inhibition to HER3-induced PTX resistance through suppressing survivin, an apoptotic inhibitor, but also an enhancement of PTX-induced apoptosis in HER2-overexpressed BC cells [84]. DOX is another chemotherapy used clinically. Significantly high level of miR-216a, miR-124-3p, miR-148/152, miR-192-5p, miR-181c, miR-381, and miR-489 have been documented to be linked with DOX sensitivity in BC cell lines [85, 86, 136–140]. Among them, miR-181c modulates drug efficiency through inactivating its downstream gene *OPN* (*osteopontin*), leading to enhanced p53-dependent transactivation and apoptosis in resistant BC cells [85]. miR-381 can enhance chemosensitization via downregulating the MAPK/FYN signaling, which ultimately overcomes drug resistance in BC patients [86]. Besides, miR-381 is associated with DDP resistance. Knockdown of miR-381 reduces DDP sensitivity to BC cell lines due to its downstream gene *MDR1* and indicates poor prognosis [87]. Additionally, miR-100 can reverse DDP-resistant BC by suppressing hematopoietic cell-specific protein 1-associated protein X-1 (HAX-1) and modulating mitochondrial apoptosis pathway [88]. Thus, a diverse range of activities mediated by miRNAs and their downstream signaling highlight the miRNAs as a regulatory entity that positively modulates the chemosensitivity in BC and suppresses tumor progression.

#### HCC

Several studies have investigated the function of miRNAs in HCC, especially patients with chemoresistance and poor prognosis. MiR-19a-3p has been identified to regulate cell proliferation and induce sorafenib resistance by modulating the PTEN/Akt pathway [89]. Conversely, miR-142-3p is reported to inhibit autophagy and enhance the sensitivity of HCC cells to sorafenib via targeting ATG5 and autophagy-related 16-like 1 (ATG16L1). This miR-142-3p-ATG5/ATG16L1 axis may be a potential therapeutic approach to reduce cyto-protective autophagy and then overcome sorafenib resistance [90]. Besides, about the DOX resistance, some miRNAs, like miR-3129-5p and miR-760, have been found to contribute to this part. The silencing of miR-3129-5p can enforce mRNA stability of *ZEB1*, a strong epithelial-mesenchymal transition-related transcription factor (EMT-TF), and this upregulation of ZEB1 finally triggers DOX resistance in HCC cells [91]. Similarly, miR-760 is positively associated with DOX sensitivity by increasing PTEN expression and decreasing the phosphorylation of Akt in HCC cell lines [92]. For platinum drugs, miR-21-5p induces resistance to DDP and promotes tumorigenesis via downregulating FAS ligand (FASLG) [93]. Besides, miR-16 facilitates the PTX resistance by targeting the IKBKB/NF-kB signaling pathway, indicating that it can be used as a treatment point [94]. Consequently, in response to chemotherapy in HCC, miRNAs appear to contribute to either tumor apoptosis or regrowth in part by various pathways related to autophagy and cell proliferation.

#### Lung cancer

DDP resistance is a severe issue in the treatment of NSCLC and many miRNAs can reverse DDP resistance. For example, as a tumor suppressor, miR-219a-5p can influence cell proliferation, cell cycle distribution and apoptosis by negatively regulating fibroblast growth factor 9 (FGF9) [95]. MiR-539 targets on doublecortin-like kinase 1 (DCLK1) and decreases cell proliferation, resulting in arrested cell cycle, more apoptosis, less invasion and migration [96]. Similarly, excessive miR-181b expression reduces CSCs-like properties and induces sensitivity to DDP via directly inactivating the Notch2/Hes1 pathway [97]. MiR-9 negatively modulates EIF5A2 and then sensitizes DDP efficiency [98]. MiR-133b attenuates the proliferation and migration abilities in DDP-resistant NSCLC cell lines via inhibiting glutathione-S-transferase P1 (GSTP1) [99]. Conversely, overexpression of miR-130b targets PTEN to trigger DDP resistance, proliferation and decreased apoptosis through the Wnt/β-catenin pathway [100]. For other drugs, like PTX, the silencing of miRNA-935 increases the expression of sex-determining region Y-box 7 (SOX7) and as a tumor suppressor, SOX7 can promote the antitumor effect of PTX, inducing cell growth arrest and apoptosis [101]. As a result, the chemoresistance regulated by miRNAs in lung cancer is linked with various pathways, affecting the cell cycle, proliferation and apoptosis.

#### CRC

Oxaliplatin resistance is a major challenge for the treatment of advanced CRC. MiR-128-3p and miR-483-3p overexpression can enhance chemosensitivity of oxaliplatin while miR-135b and miR-141 have the opposite effect [102–104, 141]. Specifically, exosomes-transmitted miR-128-3p is able to repress EMT and accelerate intracellular oxaliplatin accumulation by negatively modulating Bmi1 and MRP5 [102]. Inhibition of miR-483-3p significantly increases FAM171B in CRC cell lines and then enhances oxaliplatin resistance by reducing cell apoptosis and enforcing cell migration [103]. Conversely, miR-135b is found to be highly upregulated in CRC cell lines and patients, easily inducing oxaliplatin resistance. Anti-miR-135b can mechanically improve the expression of forkhead O1 (FOXO1) and then enhance its downstream factors Bim and Noxa, which act as core pro-apoptotic proteins in mitochondrial apoptosis. This anti-miR-135b/FOXO1/Bim/Noxa axis can sensitize CRC to therapy via increasing oxaliplatin-dependent apoptosis [104]. Another fundamental drug is 5-FU. Literatures have been documented that the upregulation of miR-195-5p and miR-181a/135a/302c improves sensitivity to 5-FU in CRC cell lines and patients [105, 106, 142]. Interestingly, miR-195-5p can either overcome stemness and chemoresistance through suppressing the Notch2/RBPJ signaling [105] or dramatically increase chemosensitivity and apoptosis via inhibiting glycerophosphodiester phosphodiesterase domain containing 5 (GDPD5) [106]. Besides, miR-148a has been proved to decrease the expression of some stem cell markers, induce apoptosis, inhibit cell invasion and migration, ultimately reduce chemoresistance in DDP-resistant CRC cell lines, partially by modulating WNT10b and β-catenin pathway [107]. These studies prove that miRNAs can serve as potential biomarkers, indicating the radiation prognosis in CRC patients.

#### Ovarian cancer (OC)

Chemotherapy is the recommended approach for curing advanced OC, especially the major drugs DDP and PTX. However, partly due to the development of drug resistance, the 5-year survival ratio keeps low. For DDP, several miRNAs have been reported to be correlated with drug efficiency. MiR-142-5p enhances DDP-induced apoptosis in OC cells by targeting multiple anti-apoptotic genes, including *X-linked inhibitor of apoptosis (XIAP), baculoviral IAP repeat-containing 3 (BIRC3), B-cell lymphoma-2 (BCL2), BCL2 like 2 (BCL2L2), and myeloid cell leukemia sequence 1 (MCL1)* [108]. MiR-378a-3p sensitizes OC cells to DDP through targeting its downstream genes *MAPK1/growth factor receptor-bound protein 2 (GRB2)* [109]. Similarly, miR-363 reduces DDP chemoresistance via modulating snail-induced EMT [110]. Besides, it has been discovered that miR-139-5p, miR-139, miR-34a and miR-330-5p also contribute to DDP chemosensitivity by inactivation of MAPK pathway and ring finger protein 2 (RNF2), inhibition of ATP7A/B, blockage of histone deacetylase 1 (HDAC1), and downregulation of S100 calcium-binding protein A7(S100A7), respectively [111–114]. Conversely, miR-149-5p aggravates DDP resistance in OC cells by directly targeting the core kinase components of the Hippo signaling pathway, STE20-like kinase (MST)1 and protein salvador homolog 1 (SAV1), leading to the inhibition of TEA domain (TEAD) transcription [115]. MiR-514 promotes DDP resistance by interacting with ATP binding cassette subfamily [116]. For PTX resistance, multiple regulatory axes have been found, including the UCA1/miR-129/ABCB1 axis, the miR-874/serine/threonine-protein kinase 2 (SIK2) axis, the miR-383-5p/TRIM27 axis, and the miR-1246/ caveolin 1 (CAV1)/p-gp/M2-type macrophage axis [117–120], which provide some new mechanistically therapeutic approaches to overcome chemoresistance and tumor progression. Thus, the pivotal double-faced roles of miRNAs in OC make them as both therapeutic candidates and direct therapeutic targets.

#### Glioblastoma (GBM)

GBM is the most common malignancy in the central nervous system and the most aggressive glioma with poor prognosis and high recurrence rates, partially resulting from chemoresistance like temozolomide (TMZ) and DDP [143]. It has been found that exosomal transmitted miR-151a improves chemosensitivity to TMZ in drug-resistant GBM cells by suppressing XRCC4-mediated DNA repair [121]. MiR-519a dramatically enhances TMZ-induced autophagy and apoptotic cell death in GBM cell lines through the targeting signal transducer and activator of transcription 3 (STAT3)/Bcl-2/Beclin-1 pathway [122]. Similarly, it has been elucidated that hypoxia-induced autophagy can affect cell mobility, tumor progression and chemosensitivity in GBM, via modulating the HIF-1α/miR-224-3p/ATG5 axis [123]. While, miR-1238 is upregulated in TMZ-resistant GBM cells, and the silencing of miR-1238 can improve chemosensitivity through directly targeting the CAV1/epidermal growth factor receptor (EGFR) pathway [124]. And for DDP treatment, miR-501-3p can sensitize GBM to DDP-induced proliferation arrest and apoptosis by targeting N-myc (MYCN) [125]. Additionally, it has been documented that upregulation of miR-29a enhances sensitivity of DDP in CD133 positive (CD133^+^) cells, a type of CSCs in GBM, and reverses poor prognosis in vitro and in vivo [144]. Taken together, miRNAs affect chemotherapy efficiency through multiple signaling pathways, including cell cycle arrest, autophagy and apoptosis, providing potential treatment targets.

#### GC

GC is the most common malignancy in Asia and is resistant to most therapies. Standard chemotherapy, like 5-FU, DDP, PTX and DOX, is the main method and first-line treatment for patients with advanced GC [145]. Various miRNAs have been discovered to be linked with the resistance to those drugs. MiR-494 is downregulated in DOX-resistant GC cells, and overexpression of miR-494 sensitizes GC cells to DOX-induced cytotoxicity by targeting the 3’untranslated region (UTR) region of phosphodiesterases 4D (PDE4D) and inhibiting its expression [126]. As the downstream of rosmarinic acid (RA), miR-6785-5p and miR-642a-3p can be reduced by RA, which enhances FOXO4 expression and reverses resistance to 5-FU [127]. Additionally, suppression of miR-17 promotes DDP or 5-FU sensitivity in GC cells by increasing death effector domain-containing DNA-binding protein (DEDD), an endogenous suppressor of tumor growth and metastasis through the EMT process [128]. MiR-193a-3p is found participating in DDP resistance through altering the levels of multiple apoptotic genes, reducing cell viability and increasing the number of apoptotic cells, all of which are related to mitochondrial apoptosis pathway [129]. Besides, exosomal delivery of miR155-5p might induce EMT and transfer cell phenotype from PTX-resistant to PTX-sensitive, via modulating GATA binding protein 3 (GATA3) and tumor protein p53-inducible nuclear protein 1 (TP53INP1) suppression, which can be a promising approach to overcome PTX resistance in GC [130]. As a result, miRNAs are observed to affect the efficacy of chemotherapy in GC cells by altering the expression profile of oncogenes, tumor suppressors, apoptotic genes or other tumor-related genes.

#### Other cancers

Gemcitabine is widely used in advanced pancreatic carcinoma. MiR-200b and miR-125a-3p are revealed to improve chemosensitivity via reversing EMT [131, 132], while miR-301 mediates gemcitabine resistance and induces EMT through downregulating cadherin 1 in pancreatic tumor cell lines [131]. In OS, DDP resistance can be alleviated by miR-340 via targeting ZEB1 [133], and miR-233 via forming the miR-233/Hsp70/JNK/JUN/ miR-233 feedback loop [134]. In CC, miR-21 is demonstrated to participate in DDP plus PTX resistance and indicates a poor prognosis [146]. What’s more, the silencing of miR-21 can rescue the expression of tumor suppressor proteins and reverse topotecan resistance in renal cell carcinoma (RCC) [147]. Additionally, in papillary thyroid carcinoma (PTC), upregulation of miR-206 contributes to euthyrox resistance by blocking the p38 and JNK signaling pathway via targeting MAP 4 K3 [135]. Thus, miRNAs play an important role in the pathways of chemotherapy response and signal transduction among various cancers by targeting different downstream genes, mainly referring to EMT, cell proliferation and apoptosis.

### MiRNAs in cancer radioresistance

Multiple factors have turned out to involve in the response of tumor cells under radiation. And most of the miRNAs are reported to positively regulate this process through different aspects, including DNA damage repair ability, cell apoptosis or autophagy, the growth-factor signal pathway, the tumor microenvironment, the immune-checkpoint, the EMT and the CSCs-like properties [148]. The miRNAs that are associated with cancer radioresistance, as well as their regulating downstream genes, are summarized in Table 2.

**Table 2:** Cancer radioresistance related miRNAs

Cancers	miRNAs	Targets/Mechanisms	References
Prostate cancer	miR-205	Disturbing DNA damage repair through PKCε and ZEB1 inhibition	[149]
	miR-875-5p	Regulating EMT; Suppressing EGFR-ZEB1 axis	[150]
	miR-124/ miR-144	Counteracting hypoxia-induced autophagy and downregulating PIM1	[151]
	miR-301a/miR-301b	Regulating miR-301a/b-NDRG2 axis	[152]
Esophageal cancer	miR-193a-3p	Reducing PSEN1	[153]
	miR-301a	Targeting WNT1, counteracting the Wnt/ β-catenin signaling pathway and reversing EMT	[154]
	miR-338-5p	Regulating survivin	[155]
	miR-17	Regulating C6orf120	[156]
	miR-98	Binding to BCL-2	[157]
	miR-124	Regulating miR-124/ CDK4 axis	[158]
Nasopharyngeal carcinoma	miR-495	Binding the 3’UTR of GRP78 and inhibiting its expression	[159]
	miR-24-3p	Modulating 3’UTR and 5’UTR of Jab1/CSN5	[160]
	miR-24	Binding SP1	[161]
	miR-150	Inhibiting GSK3β	[162]
	miR-222	Interacting with PTEN	[163]
	miR-19b-3p	Activating TNFAIP3/NF-κB pathway	[164]
Cervical cancer	miR-4778-3p	Negatively regulating NR2C2 and Med19 expression; Enhancing apoptosis-related genes expression	[165]
	miR-15a-3p	Promoting tumor protein D52	[166]
	miR-125	Downregulating CDKN1A and targeting p21	[167]
	miR-424	Targeting aprataxin	[168]
	miR-21	Decreasing PTEN and increasing p-Akt/HIF-1α; Inhibiting autophagy via Akt-mTOR pathway	[169]
Lung cancer	miR-198	Inhibiting HGF/c-MET pathway	[170]
	miR-99a	Binding to mTOR	[171]
	miR-558	Associating with *AATK*	[172]
	miR-148b	Modulating MutL homologue 1	[173]
	miR-335	Reducing the expression of PARP-1, downregulating NF-κB	[174]
	miR-1246	Inhibiting DR5	[175]
	miR-21	Upregulating HIF-1α	[176]
	miR-208a	Targeting p21 with a corresponding activation of the Akt/mTOR pathway	[177]
Oral cancer stem cells	miR-218	Activating miR-218/Bmi1 axis	[178]
Hepatocellular carcinoma	miR-92b	Reducing p57kip2 expression	[179]
Breast cancer	miR-142-3p	Attenuating CSCs characteristics and inhibiting β-catenin expression	[180]
	miR-22	Negatively modulating Sirt1	[181]
	miR-668	Forming miR-668/ IκBα/NF-κB axis	[182]
Ovarian cancer	miR-214	Depressing PETN and consequently activating PI3K/Akt pathway	[183]

#### PC

Radiotherapy is an adjuvant treatment of surgery in PC. However, radioresistance has been gradually reported and results in poor prognosis. It has been observed that miR-205 is responsible for dramatically improving radiation sensitivity in vitro and in vivo by disturbing DNA damage repair through PKCε and ZEB1 inhibition [149]. MiR-875-5p is able to inhibit EMT and enhance radiation efficiency via suppression of the EGFR-ZEB1 axis [150]. Similarly, overexpression of miR-124 or miR-144 can promote radiosensitivity in PC cells through counteracting hypoxia-induced autophagy and downregulating PIM1 [151]. While, miR-301a and miR-301b show the opposite effect. As hypoxia-responsive miRNAs, they are highly expressed under hypoxia in PC cells and can increase autophagy by reducing *NDRG2*, a tumor suppressor gene in PC. And this miR-301a/b-NDRG2 axis on autophagy and radiosensitivity of PC can be a promising therapeutic point [152]. Therefore, it is apparent that miRNAs regulate radiotherapy in PC through multiple methods, which, in turn, cause aberrant translational inhibition or degradation of their target mRNAs, and further studies to validate the clinical utility of miRNAs among PC patients are warranted.

#### Esophageal cancer (EC)

EC is one of the leading causes of death among malignancies, and resistance to radiotherapy is proved to be a big challenge during treatment. It has been found that overexpression of miR-193a-3p can induce radioresistance by reducing PSEN1 [153]. In contrast, some miRNAs are correlated to radiosensitivity. MiR301a enhances radiosensitivity and suppresses tumor cell migration via targeting WNT1 in EC, thus counteracting the Wnt//β-catenin signaling pathway and reversing EMT [154]. Similarly, miR-338-5p can improve radiosensitivity in vitro and in vivo through survivin [155]. MiR-17 is documented to highly sensitize radioresistant cells to X-ray radiation by regulating C6orf120, especially in CSCs [156]. MiR-98 is downregulated in radioresistant cell lines and overexpression of miR-98 can increase radiosensitivity by preventing tumor growth and resistance tolerance via directly binding to BCL-2 [157]. Besides, the miR-124/cyclindependent kinase 4 (CDK4) axis has been identified to decrease tumor aggression and aggravation, by triggering apoptosis in EC cell lines, which ultimately enhances radiotherapy efficiency [158]. Altogether, miRNAs affect the radiation efficacy in EC by mediating multiple pathways, including cell proliferation, migration, apoptosis and autophagy, supporting the idea that miRNAs can serve as potential targets to overcome the radioresistance in EC.

#### NPC

Radiotherapy is the standard therapy for patients with NPC. However, the development of radioresistance limits efficiency and leads to poor prognosis. It has been demonstrated that miR-495 can bind the 3’UTR of glucose-regulated protein 78(GRP78) and inhibit its expression, which might be involved in EMT phenotype transition and then enhance radiotherapy efficacy [159]. MiR-24-3p can suppress tumor growth and increase radiosensitivity by directly modulating 3’UTR and 5’UTR of Jab1/CSN5, and this miR-24/Jab1/CSN5 axis can serve as prognostic markers for NPC recurrence [160]. Beside, miR-24 is able to bind another downstream gene, *specificity protein 1* (*SP1*), which is found to be associated with cell viability as well as radiosensitivity in NPC cells, and the miR-24/SP1 pathway can reduce radioresistance in vitro and in vivo [161]. While some miRNAs have been found to contribute to radioresistance in NPC. Overexpression of miR-150 is discovered in radioresistant NPC cells and tissues, which can inhibit GSK3β and trigger therapy resistance [162]. MiR-222 interacts with PTEN in NPC cells, resulting in elevated cell proliferation, colony formation and finally radiation resistance [163]. Additionally, miR-19b-3p can decrease sensitivity to irradiation in vitro and in vivo by activating the TNFAIP3/NF-κB pathway, which might serve as a potential therapeutic point in NPC treatment [164]. Taken together, miRNAs play controversial roles in response to radiation, and it is possible that by targeting some core miRNAs, patients with NPC can develop less resistance and benefit more from radiotherapy.

#### CC

Radiotherapy is an essential approach for locally advanced CC. However, around 30% of radiotherapy-treated patients have been found occurring recurrence or even metastasis, partially resulting from radioresistance. It has been reported that miRNAs participate in either radiation responsiveness or radioresistance. For example, miR-4778-3p can decrease the vitality, proliferation, migration of radioresistant CC cells and modulate radiosensitivity of CC by negatively regulating nuclear receptor subfamily 2 group C member 2 (NR2C2) and Med19 expression, as well as enhancing expression of apoptosis-related genes, like *Bax, Caspase-3, Caspase-8, and Caspase-9* [165]. MiR-15a-3p is downregulated in CC cell lines and tissues, and increasing level of miR-15a-3p can enhance radiosensitivity by promoting tumor protein D52, which decreases cell proliferation and increases cell apoptosis [166]. Similarly, miR-125 sensitizes CC cells to radiation treatment via downregulating CDKN1A and targeting p21 [167], and miR-424 modulates radiosensitivity through targeting aprataxin [168]. In contrast, miR-21 induces radioresistance in CC and overexpression of miR-21 can result in decreased PTEN and increased p-Akt/HIF-1α. MiR-21 is also found to inhibit radiation-induced autophagy via the Akt-mTOR pathway, which leads to radioresistance [169]. Thus, this complicated relationship between miRNAs and other downstream pathways represents a promising target for conquering radioresistance in CC.

#### Lung Cancer

Multiple miRNAs have been associated with radiation efficacy in NSCLC, including miR-198, miR-99a, miR-558 and miR-148b [170–173]. To be specific, overexpression of miR-198 is found to suppress cell proliferation, migration, invasion and promote apoptosis by inhibiting the hepatocyte growth factor (HGF)/c-MET pathway, which overcomes resistance to radiation in NSCLC cells and tissues [170]. MiR-99a is identified to directly bind to mTOR and functions as a tumor suppressor, modulating radiosensitivity [171]. Similarly, it has been discovered that miR-558 is associated with *apoptosis-associated tyrosine kinase* (*AATK*), a radiosensitization-associated gene, and regulates the development of resistance to radiotherapy [172]. The expression of miR-148b is dramatically decreased in both serum and tumor tissues of radioresistant lung cancer patients, and the upregulation of miR-148b can reverse radioresistance by modulating MutL homolog 1 (MLH1), which indicates that miR-148b can serve as a potential biomarker of response to radiation [173]. And in SCLC, it has been reported that elevated miR-335 reduces the expression of poly [ADP-ribose] polymerase 1 (PARP-1) on both mRNA and protein level, correspondingly resulting in the downregulation of NF-κB protein level, thus modulating radiosensitivity of SCLC [174]. In contrast, several miRNAs have been demonstrated to induce radioresistance. Extracellular miR-1246 can promote proliferation and radioresistance in lung cancer cells by inhibiting death receptor 5 (DR5) [175]. MiR-21 is able to link with HIF-1α, and the upregulation of HIF-1α promotes the core enzymes of glycolysis. This miR-21/ HIF-1α/glycolysis signaling can trigger resistance to radiation in cancer cells [176]. Additionally, overexpression of miR-208a enhances cell proliferation and increases radioresistance by targeting p21 with a corresponding activation of the Akt/mTOR pathway in lung cancer cells, as well as inhibiting apoptosis via cell cycle distribution arrest [177]. As a result, miRNAs can be used as potential candidates to develop novel approaches to reverse lung cancer cell resistance to radiotherapy.

#### Other cancers

There are still multiple miRNAs implicated in the response of other cancer types to radiation. In oral cancer stem cells (OCSCs), activation of the miR-218/Bmi1 axis can inhibit tumor growth and enhance radiosensitivity [178]. In HCC, miR-92b reduces p57kip2 expression in HCC cell lines and tissues, then promoting the radioresistance to ionizing radiation (IR)-based radiotherapy [179]. And in BC, miR-142-3p can reduce radioresistance by attenuating the characteristics of CSCs and inhibiting β-catenin expression [180]. Similarly, miR-22 prevents tumorigenesis and improves radiosensitivity by negatively modulating Sirt1 [181]. On the contrary, miR-668 triggers radioresistance by forming the miR-668/ IκBα/NF-κB axis. MiR-668 overexpression decreases the IκBα level, increases NF-κB, and then enhances radioresistance in BC cell lines [182]. Besides, in OC, IR upregulates the expression of miR-214 in vitro and in vivo, which depresses PETN and consequently activates the PI3K/Akt pathway, resulting in radioresistance [183]. Taken together, these studies provide fresh insight into the contribution of miRNAs in multiple cancers radiation resistance, and it is essential to identify more miRNAs that can be potential therapeutic targets for radioresistant cancer patients.

### CircRNAs and cancer therapy resistance

CircRNAs are a novel group of endogenous noncoding RNAs, defined by their typical circular covalently bonded structure without a 5′ cap or a 3′ Poly A tail. They can functionally compete with endogenous RNAs or miRNA sponges, mediate gene transcription and even translation. Based on their high conservation, abundance and tissue specificity, circRNAs can serve as special molecular markers in the disease, including caners [184]. Accumulating evidences have been demonstrated their roles in cancer development, especially in therapeutic resistance [185]. In this part, we systematically emphasize the literatures on circRNAs involving in chemoresistance and radioresistance.

### CircRNAs in cancer chemoresistance

Some circRNAs have been reported to promote chemoresistance. In lung cancers, for instance, hsa_circ_0003998 is found to be overexpressed in lung adenocarcinoma (LAD) tissues and DOX-resistant cell lines. The silencing of hsa_circ_0003998 can increase chemotherapy sensitivity, suppress proliferation and promote apoptosis by targeting miR-326 in LAD cells [186]. Hsa_circ_0004015 enhances the resistance of NSCLC to gefitinib via sponging miR-1183 and targeting its downstream gene *PDPK1* [187]. Similarly, circ_0076305 is demonstrated to regulate DDP resistance in NSCLC through positively mediating STAT3 by binding to miR-296-5p [188]. Besides, in OS, circPVT1 is discovered to be significantly upregulated in the tumor tissues, serum and chemoresistant cell lines. Knockdown of circPVT1 can reduce resistance to DOX and DDP in OS cells via decreasing *ABCB1*, a canonical drug resistance-related gene [189]. Also, hsa_circ_0081001, screened by the RNA sequencing, has been found to be upregulated in OS cell lines, tissues and serum, linked with poor overall survival and chemoresistance [190, 191]. And the hsa_circ_0001258/hsa-miR-744-3p/glutathione S-transferase Mu 2 (GSTM2) axis has been reported to contribute to chemoresistance in OS [190, 191]. Additionally, circ_0004674 is remarkably increased in OS chemoresistant cells and tissues, involving in the circRNA-miRNA-mRNA pathway and indicating poor prognosis [192]. Moreover, in liver cancer, circβ-catenin, highly related to malignant phenotypes in vitro *and* in vivo, can modulate tumor cell proliferation by activating the Wnt signaling [193]. In BC, the circ_0006528/miR-7-5p/Raf1 axis is identified in human tissues and ADM-resistant cells, linked with BC chemoresistance [194, 195]. And inhibition of circ-CDR1as can increase the sensitivity of 5-FU-resistant BC cells via the miR-7/CCNE1 axis [196]. As for GC, hsa_circ_0081143 can adjust the miR-646/CDK6 pathway and promote DDP resistance [197]. And hsa_circ_0000199 is identified to play an important role in DDP resistance by sponging miR-198 and promoting PIK3R1 expression [198]. In acute myeloid leukemia (AML), circPAN3/miR-153-5p/miR-183-5p/XIAP axis is reported to mediate drug resistance [199]. What’s more, in bladder cancer, circELP3 participates in DDP resistance, which provides a new insight into drug resistance [200]. As a result, multiple circRNAs have been proved to enhance chemoresistance in different cancers, which can function as knockdown or inhibiting target in further investigations.

In contrast, several circRNAs may have the ability to attenuate chemoresistance. For example, in PC, circFoxo3 has been reported to suppress tumor progression and aggravation by upregulating Foxo3 and downregulating EMT [201]. In BC, circRNA-MTO1 is able to reverse chemoresistance through the TRAF4/Eg5 axis [202], and hsa_circ_0025202 is found to improve TAM sensitization by affecting miR-182-5-p, along with further regulating the expression of FOXO3a [203]. Additionally, circKDM4C is proved to be decreased in BC patients, suggesting poor prognosis. It can significantly inhibit BC proliferation, metastasis, and DOX resistance in vitro and in vivo, by sponging miR-548p and targeting the downstream PBLD, a functional tumor suppressor in BC. This cirKDM4C/miR-548p/PBLD axis indicates a promising therapeutic approach for patients [204]. In OC, unlike the negative effect of circ-CDR1as on BC chemotherapy, overexpression of circ-CDR1as can enhance DDP-induced cell apoptosis via modulating suppressor of cancer cell invasion (SCAI) and miR-1270, and this Cdr1as/miR-1270/SCAI axis is responsible for sensitizing OC to DDP [205]. Therefore, it is plausible that circRNAs also contribute to chemosensitivity in multiple cancers by preventing tumorigenesis and metastasis.

### CircRNAs in cancer radioresistance

Recently, circRNAs are also proved to be associated with cancer radioresistance. In EC, for example, circVRK1 is found to be downregulated while overexpression of it can inhibit cell proliferation, migration, and EMT, resulting in reversed radioresistance by miR-624-3p/ PTEN/PI3K/Akt signaling pathway [206]. In GBM, circ-AKT3 is reported to encode AKT3-174aa, a novel tumor suppressor protein, and enhance radiation sensitivity cells by negatively regulating the PI3K/Akt pathway [207]. Besides, high-throughput sequencing and bioinformatic analysis reveals significant circRNAs expression in cancer radioresistance, like hsa_circ_0004015 [208], circRNA_001059 and circRNA_000167 [209] circATP8B4 [210], hsa_circ_0000734 [211], etc., which establishes the circRNA-miRNA-target gene interaction network. One circRNA can target various miRNAs, and one miRNA can also suppress its downstream genes. In their study, hsa_circ_0004015 is linked with hsa-miR-3163, hsa-miR-3065-5p, hsa-miR-551b-5p, hsa-miR-4311, and hsa-miR-875-3p, and these different miRNAs may be associated with their downstream genes, including *ZYX, PRKAR1A, BTAF1, LRP3, and ETS1*, which contributes to radioresistance [208]. CircRNA_001059 and circRNA_000167 are another two largest nodes in this network and have been found in radioresistant EC cells [209]. CircATP8B4 is one of the circRNAs in extracellular vesicles isolated from radioresistant glioma cells and induces therapy resistance by sponging U251 miR-766 [210]. Hsa_circ_0000734 is discovered to modulate hsa-miR-432-5p and hsa-miR-124-5p, which can further control the expression of target genes [211]. As a result, circRNAs play a comprehensive role in tumor radiotherapy, which provides some new insights on therapeutic approaches. However, the deeper mechanism among this circRNA-miRNA-target gene interaction network is not clear, calling for more investigations.

### Other ncRNAs and cancer therapy resistance

In recent years, a growing body of literatures suggest that other ncRNAs, such as snRNAs, snoRNAs, piRNAs, and siRNAs, are responsible for cancer development and therapy efficiency. Among them, it is now widely appreciated that siRNAs, normally used in combination with drugs, play a substantial role in improving progression-free and/or overall survival. These siRNA-drug complexes can be achieved by nanotechnology-based delivery systems, along with controlled release and delivery. For instance, autophagy has been found to serve as a pro-survival contributor to chemotherapy resistance in some cancer cells. Diverse factors, like the autophagy initiation factor *Beclin1*, regulate the activity of autophagy through signaling in the periphery and within the tumor, thus promoting tumorigenesis and leading to chemotherapy resistance. Based on that, a self-assembled nano-prodrug platform has been developed with the synergistic effect of DDP and *Beclin1* siRNA to fight against DDP-resistant lung cancer. The blockage of *Beclin1* facilitates the long-term use of DDP in vivo, significantly augmenting its therapeutic efficacy and clinical outcome [212]. Besides, cytosolic Ca^2+^ is a crucial signal transduction regulator, and abnormal expression or activities of transmembrane Ca^2+^ channels or pumps have been proved to be associated with tumorigenesis and tumor progression. As a result, an alternative strategy, which is through the combinatorial delivery of Ca^2+^ channel siRNA with cytotoxic drugs like DOX, has been tested to overcome MDR in BC. Both in vitro and in vivo data have shown that the DOX/Ca^2+^ channel siRNA cocktail co-delivery presents good biocompatibility and high efficiency for conquering MDR and enhancing cancer therapy with nanoformulations [213]. Despite Ca^2+^ channel, overexpression of drug efflux transporters, like pgp protein, is another key mechanism for MDR in tumor cells. Literatures have shown that *pgp* gene encodes *MDR1*, which contributes to the formation of a drug efflux pump that inhibits the intracellular buildup of drugs. Herein, by using mesoporous silica nanoparticles (MSNP), DOX and *pgp* siRNA can be functionally packaged together and effectively co-deliver to a drug-resistant BC to promote cytotoxicity in an additive or synergistic manner [214]. Therefore, the utilization of siRNA in combination with chemotherapy enhances the therapeutic effect via mainly targeting autophagy and MDR, which ultimately suppresses tumor regrowth and augments treatment sensitivity. However, about the role of siRNA in radiotherapy, more investigations for future application are warranted.

Similar to siRNAs, piRNAs have also received increased attention as they collaborate with downstream factors, altering tumor development and progression. For example, the effect of piRNA 54,265 (piR-54,265) has been described in some studies. In CRC, an increased serum level of piR-54,265 correlates with poor prognosis and outcome, even with resistance to anticancer agents in patients. The biochemical assays verify that it can specifically bind to PIWIL2, facilitating the formation of PIWIL2/STAT3/phosphorylated-SRC complex and phosphorylation of STAT3, thus enhancing proliferation and aggravation of CRC cells. And the blockade of piR-54,265 leads to increased survival of mice bearing CRC [215]. In addition, piR-39,980, overexpressed in neuroblastoma cells, serves as an oncogene and contributes to tumor progression, whereas its suppression results in decreased viability, invasion, and migration of cells. It has been documented that the downregulation of piR-39,980 triggers senescence of tumor cells, which consequently ameliorates the sensitivity of DOX and boosts drug-induced apoptosis [216]. Conversely, piR-36,712 is reported as a novel tumor suppressor in BC. Functional studies suggest that the lower level of piR-36,712 results in higher *SEPW1* expression, which may inhibit P53, lead to the increased Slug but decreased P21 and E-cadherin levels, and ultimately promote cancer cell proliferation, invasion and migration. Based on that, piR-36,712 can synergistically facilitate the anticancer effects of PTX and DOX [217]. Overall, in response to chemotherapy, most piRNAs appear to contribute to tumor regrowth and resistance to therapy, partially by inducing proliferation and migration, while some piRNAs are identified as the tumor suppressor and have potential clinical value. Nevertheless, it warrants more researches to further elucidate the effect of piRNAs on radiotherapy.

Although little is known with respect to the functions of other ncRNAs like snRNAs and snoRNAs in human cancer, emerging evidences have indicated that they may play an important role in cancer development, migration, and aggravation, and may act as diagnostic and prognostic biomarkers. Regarding the snRNAs, studies have revealed that fragments derived from U2 snRNA (termed RNU2-1f) are more abundant in serum of patients with different tumors, including CRC, pancreatic cancer [218], OC [219], lung cancer [220], metastatic melanoma [221], et al., and even in cerebrospinal fluid of patients with primary central nervous system lymphoma [222]. Besides, diverse snoRNAs have been proved to serve as biomarkers in different tumors. For example, SNORD33, SNORD66, and SNORD76 present high plasma expression in NSCLC patients and can provide potential markers for early detection [223]. SNORA21 is upregulated in CRC and promotes cancer progression, acting as a key oncogenic snoRNA to enhance cell proliferation and invasion via regulating multiple cancer-related pathways [224]. Even though a panel of snRNAs and snoRNAs that associates with tumors development have been identified, the role of them in therapy resistance is still unclear. It is possible that with more investigations, they can not only be used as a noninvasive diagnostic and prognostic tool but also serve as the fresh therapeutic targets.

### RNA modifiers and cancer therapy resistance

Post-transcriptional modifications of RNA are very common and usually make a profound impact on RNA biological function. There has been a widespread understanding of the modifications in mRNA, including splicing, transportation, translation, degradation and other processes [225]. With the development of technology and research, the area of ncRNA modification has been gradually explored, especially in lncRNAs, miRNAs, and circRNAs, like m6A [226], uridine [227], and pseudouridine [228]. Among them, m6A, also named as *N*^6^-methyladenosine, a methylation modification on the sixth N atom of base A, is one of the most abundant methylation modifications in eukaryotes. The identification of m6A, along with its writers, readers, and erasers, has brought the RNA modification into epitranscriptomics [226]. 3′ RNA uridine, the non-templated addition of uridine(s) at the end of the RNA, is also a fundamental mediator in epitranscriptomics. It is mediated by Terminal Uridylyl Transferases (TUTases) and can functionally regulate both mRNAs and ncRNAs [227]. Pseudouridine (ψ) is the most abundant RNA modification. It is an isomer of the conventional RNA nucleoside uridine and is catalyzed by perturbing pseudouridine synthases (PUS) from uridine. The incorporation of ψ nucleoside can enhance the base accumulation ability of RNA and make the sugar-phosphate skeleton more rigid [228]. Currently, more and more RNA modifiers, especially m6A, have been revealed to contribute to disease progression, including cancer therapy resistance.

### LncRNA modifiers and therapy resistance

Different modifiers, especially m6A, play a vital role in lncRNAs modification in tumor cells and lead to therapy resistance. For example, m6A related modification can enhance the function of lncRNA through RNA processing, transport and stability. It has been reported that m6A-induced lncRNA RP11 can promote the progression and metastasis of CRC via post-translational upregulation of ZEB1 [229]. High level of lncRNA RP11 also reduces the cytotoxicity of DDP [230] and erlotinib [231], triggering chemotherapy resistance in tumor cells. Besides, m6A has been found to be significantly enriched within lncRNA FAM225A and increase its RNA stability. Then FAM225A functionally acts as a ceRNA for sponging miR-590-3p and miR-1275, leading to the overexpression of its downstream gene integrin β3 (ITGB3), and activating of FAK/PI3K/Akt signaling. All of these enhance proliferation and aggravation of NPC, resulting in therapeutic resistance [232]. LncRNA MALAT1 is a well-known tumor-related lncRNA [233]. It has been found that methyltransferase-like protein 16 (METTL16), a m6A encoder, can specifically bind to the triple helix region of MALAT1 and this METTL16-MALAT1 complex might associate with the oncogenic activity of MALAT1 [234]. M6A modification can also trigger a local change in the structure of MALAT1, enhance its binding with heterogeneous nuclear ribonucleoprotein C (HNRNPC) and induce “m6A-switch” [235]. Moreover, m6A modification can degrade lncRNA and make tumor cells resistant to treatment. LncRNA GAS5 has been proved to be a tumor suppressor in many studies via directly sponging miR-21 and enhancing chemotherapy sensitivity. It affects DDP resistance to CC by regulating Akt phosphorylation [236]. It has been demonstrated that m6A induces degradation of GAS5 through m6A reader protein YTHDF2 [237] and YTHDF3 [238], which increases therapy resistance and results in poor prognosis. Except for m6A, ψ has been linked with some tumor-related lncRNAs, including MALAT1 and RN7SK [239]. However, there still need more investigations to dissect underlying mechanisms and function of ψ, as well as other modifiers, in the relationship between lncRNAs and tumor resistance to therapies.

### MiRNA modifiers and therapy resistance

Similar to lncRNAs, m6A related modification has been found to involve in miRNA synthesis. As the precursor of miRNA, pri-miRNA needs to be transformed into pre-miRNA with the assistance of DGCR8 and Drosha. And then, pre-miRNA will be exported to the cytoplasm. In the cytoplasm, pre-miRNA can be cut into mature miRNA by Dicer. During this process, METTL3 has been reported to methylate pri-miRNA, label it for DGCR8 recognition and processing, and in the end promote miRNA maturation [240]. This mechanism has been discovered in different tumor cells. In bladder cancer cells, METTL3 can interact with DGCR8 and positively regulate the pri-miR-221/222 synthesis in an m6A-based pathway, which might provide new insight on bladder cancer therapy and therapy resistance [241]. MiR-221/222 has been also upregulated in myeloma, especially in patients with bad prognosis. Functionally, ATG12 is proved to be the downstream gene of miR-221/222, and overexpression of miR-221/222 can suppress autophagy via directly binding to ATG12 and the p27^kip^(p27)- mTOR signaling. This miR-221/222-ATG12/p27-mTOR autophagy-regulatory axis correlates with dexamethasone resistance in myeloma, resulting in tumor progression and aggravation [242]. In CRC, METTL3 is linked with pri-miR-1246 and targets its downstream anti-oncogene SPRED2. This METTL3/miR-1246/SPRED2 axis promotes tumor development and metastasis, possibly inducing treatment resistance [243]. The same as METTL3, METTL14 can interact with DGCR8 and positively modulate the pri-miR-126 through m6A, thus affecting the role of miR-126 on drug resistance [244, 245]. Besides, as the reader of m6A, Heterogeneous Nuclear Ribonucleoprotein A2/B1 (HNRNPA2/B1) can recognize pri-miRNA and push Drosha processing to generate pre-miRNA. Overexpression of HNRNPA2/B1 upregulates miR-1266-5p, miR-1268a and miR-671-3p, downregulates miR-29a-3p, miR-29b-3p and miR-222, and reduces sensitivity to TAM and fulvestrant in BC cell lines [246]. Uridine related modification has been also gradually reported in miRNAs, like the LIN28/let-7 pathway. After pre-let-7 being exported to the cytoplasm, LIN28A marks the terminal loop of pre-let-7 and recruits TUTase ZCCHC11 that polyuridylates pre-let-7. This modification inhibits miRNA synthesis and promotes tumorigenesis, which is also related to various tumor treatment resistance, including radiotherapy resistance in lung cancer and PC, chemotherapy resistance in liver cancer, BC and OC [247]. Emerging evidences have been shown that those modifiers in miRNAs provide some fresh therapeutic benefit in cancer, and raise new hope to patients, especially with therapy resistance.

### CircRNA modifiers and therapy resistance

As a newly discovered ncRNA, circRNAs play an important role in the development of tumor cells. For instance, circNSUN2 has been found highly increased in tumor tissues and serum samples from CRC patients with liver metastasis and usually indicated poor prognosis. Specifically, m6A modification promotes circNSUN2 transport from the nucleus into the cytoplasm and then forms a circNSUN2/IGF2BP2/HMGA2 RNA-protein ternary complex in the cytoplasm. With the help of this complex, circNSUN2 is able to enhance the stability of HMGA2 mRNA and induce CRC aggravation [248]. HMGA2 can also decrease the chemotherapy sensitivity and immunotherapy efficiency in different tumor cells via other signaling pathways [249, 250]. Besides, m6A modification promotes the translation of circRNA. It has been discovered that m6A motifs are enriched in circRNAs and initiate translation with the initiation factor eIF4G2, m6A reader YTHDF3 and enhancer METTL3/14 [251]. This indicates a potential role of circRNA-derived proteins in response to stress and pressure, while whether this effect can regulate therapy resistance in tumor still need more exploration.

## Conclusion

NcRNAs, along with the modifiers, are the crucial mediator of tumorigenesis and development and play a fundamental role in tumor resistance to therapies by targeting multiple downstream genes or signaling pathways, interfering with apoptosis, proliferation, autophagy and tumor migration, inducing CSCs-like properties and affecting EMT, which can be used as the potential targets in ncRNAs/modifiers-based therapeutic interventions.

### Perspectives

With the development of biotechnologies like high-throughput sequencing, bioinformatic analysis, genome modification, mouse modeling and pharmaceutical chemistry, functional studies of ncRNAs/modifiers are able to provide some novel perspectives against cancer. On the one hand, their expression patterns are significantly different in serum from tumor patients, indicating their roles as the biomarkers to reflect tumor stage and disease progression. On the other hand, several ncRNAs, lncRNAs and miRNAs in particular, correlate to therapies efficiency and prognosis, causing resistance/sensitivity to chemotherapy or radiotherapy. Various genes have been proved in mouse models by using double-stranded RNA-mediated interference (RNAi) and single-stranded antisense oligonucleotides (ASOs). For example, inhibiting MALAT1 with ASO can trigger differentiation and suppress metastasis in mice bearing BC [252, 253]. Also, targeting MALAT1 by ASO can inhibit metastasis in a lung cancer xenograft model [254]. Based on the exciting results from in vivo experiments, ncRNA carriers or systems have been proposed and substantially investigated, including nanoparticles, ncRNA modification, and oncolytic adenovirus strategy. With advances in these delivery systems, several clinical trials on ncRNA-guided precision medicine have been conducted or are ongoing (https://clinicaltrials.gov). Among current candidates, miRNAs are the most widely studied, and some of them have already reached phase 3 or 4, such as miR-31-3p and miR-31-5p in CRC [255], miR-21 and miR-200 in oral cancer [256, 257]. Besides, lncRNAs and circRNAs are also recruited into trials, including MALAT1 in BC [252, 253] and lung cancer [254], HOTAIR in thyroid cancer [258]. In addition, siRNAs, normally loaded with lipid nanoparticles, have been applied into clinical tests and some siRNA-based drugs have already accomplished phase 2 clinical trials, such as DCR-MYC for *MYC* knocking down to arrest the cell cycle in HCC, Atu027 for *PKN3* knocking down to modulate cell migration in metastatic pancreatic adenocarcinoma, etc. [3]. Accordingly, ncRNAs, as well as the modifiers, can be probable targets to estimate tumor therapies response, therefore contributing to the development of individualized treatment design.

Nevertheless, challenges persist before these techniques being broadly implemented into clinics. First, due to the variation of ncRNAs in length and modes of action, as well as the complicated roles of ncRNAs/modifiers within different tumors, it is difficult to choose the proper and specific target from numerous candidates, which requires further understanding and evaluation on genomic and functional approaches for basic and translational research. Second, even with a perfect target, it is not easy to get a desirable delivery strategy with a high binding affinity. The constituent of the tumor microenvironment is heterogeneous, making the delivery and application of ncRNAs difficult, such as low transfection efficacy, off-target effects, a short half-life due to RNA instability and degradation. To solve this problem, the current delivery system is expected to be improved in subsequent studies. The combination of multiple carriers may be also an alternative, like a combination of nanoparticles with tissue-specific receptors, facilitating the precise transportation of target. Besides, another critical issue is how to ameliorate the actual bioavailability within the tumor tissues while diminishing the cytotoxicity to normal tissues. A reasonable measurement should be developed to calculate the relative efficiency and ensure high quality. Last but not least, the majority of researches illustrating functions of ncRNAs/modifiers are currently preclinical, and most of them are limited to a particular tumor type or therapeutic modality. Once getting the appropriate gene candidate as well as the consistent delivery carrier, more efforts should be focused on assessing patients’ response to ncRNA/modifier-related treatment in clinical studies in order to improve the understanding of long-term influence and adverse effects that have never been measured before. Except for these problems, ncRNAs/modifiers, serving as either oncogenes or tumor suppressors, might provide remarkable potential therapeutic approaches for therapy-resistant patients to overcome resistance and increase survival in the future.

## Data Availability

Please contact the corresponding author for all data requests.

## Abbreviations

5-FU: 5-fluorouracil
AATK: Apoptosis-associated tyrosine kinase
ABC: ATP-binding cassette
ABCC1: ATP-binding cassette subfamily c member 1
AML: Acute myeloid leukemia
ARSR: Activated in renal cell carcinoma with sunitinib resistance
ASOs: Single-stranded antisense oligonucleotides
ATG16L1: Autophagy-related 16-like 1
ATG7: Autophagy-related 7
BC: Breast cancer
BCAR4: Breast cancer antiestrogen resistance 4
BCL2: B-cell lymphoma-2
BCL2L2: BCL2 like 2
BIRC3: Baculoviral IAP repeat-containing 3
BLACAT1: lncRNA bladder cancer-associated transcript 1
CAV1: Caveolin 1
CC: Cervical cancer
CCAT2: Colon cancer associated transcript 2
CDK4/6: Cyclin dependent kinase 4/6
CDKN1A: Cyclin-dependent kinase inhibitor 1A
ceRNA: Competing endogenous RNA
circRNAs: Circular RNAs
CRC: Colorectal cancer
CRNDE: Colorectal neoplasia differentially expressed
CSCs: Cancer stem cells
DCLK1: Doublecortin-like kinase 1
DDP: Cisplatin
DEDD: Death effector domain-containing DNA-binding protein
DGCR5: DiGeorge syndrome critical region gene 5
DOX: Doxorubicin
DR5/8: Death receptor 5/8
DYRK1A: Dual-specificity tyrosine-(Y)-phosphorylation regulated kinase 1A
EC: Esophageal cancer
EGFR: Epidermal growth factor receptor
EIF4A2/5A2: Eukaryotic initiation factor 4A2/5A2
EMT: Epithelial-mesenchymal transition
EMT-TF: Epithelial mesenchymal transition related transcription factor
ER: Estrogen receptor
FASLG: FAS ligand
FGF9: Fibroblast growth factor 9
FOXO1/4: Forkhead box O1/4
GAS5: Growth arrest special 5
GATA3: GATA binding protein 3
GBM: Glioblastoma
GC: Gastric cancer
GDPD5: Glycerophosphodiester phosphodiesterase domain containing 5
GRB2: Growth factor receptor-bound protein 2
GRP78: Glucose-regulated protein 78
GSKIP: GSK3β interaction protein
GSTM2: Glutathione S-transferase Mu 2
GSTP1: Glutathione-S-transferase P1
HANR: HCC associated long non-coding RNA
HAX-1: Hematopoietic cell-specific protein 1-associated protein X-1
HCC: Hepatocellular carcinoma
HDAC1: Histone deacetylase 1
HER2: Human epidermal growth factor receptor 2
HGF: Hepatocyte growth factor
HIF: Hypoxia-inducible factor
HK2: Hexokinase-2
hnRNA: Pre-messenger RNA
HNRNPA2/B1: Heterogeneous Nuclear Ribonucleoprotein A2/B1
HNRNPC: Heterogeneous nuclear ribonucleoprotein C
HOTAIR: lncRNA HOX antisense intergenic RNA
HOTTIP: lncRNA HOXA transcript at the distal tip
HOXA1: Homeobox A1
IER3: Immediate early response 3
IGF-1R: Insulin-like growth factor 1 receptor
IR: Ionizing radiation
ITGB3: Integrin β3
LAD: Lung adenocarcinoma
LEIGC: Specifically downregulated lncRNA in GC
Lnc TINCR: Terminal differentiation-induced non-coding RNA
lnc-ATB: lncRNA activated by TGF-b
lncRNA HULC: Highly upregulated lncRNA in HCC
lncRNA MALAT1: Metastasis-associated lung adenocarcinoma transcript 1
LncRNA NNT-AS1: lncRNA nicotinamide nucleotide transhydrogenase-antisense RNA1
lncRNAs: Long non-coding RNAs
MAPK: Mitogen-activated protein kinase
MCL1: Myeloid cell leukemia sequence 1
MDR: Multi-drug resistance
MEG3: lncRNA maternally expressed gene 3
METTL16: Methyltransferase-like protein 16
miRNAs: microRNAs
MLH1: MutL homolog 1
MRP1: Multidrug resistance-associated protein 1
MSNP: Mesoporous silica nanoparticles
MST: STE20-like kinase
mTOR: Mammalian target of rapamycin
MTX: Methotrexate
MYCN: N-myc
ncRNAs: Non-coding RNAs
NPC: Nasopharyngeal carcinoma
NR2C2: Nuclear receptor subfamily 2 group C member 2
OC: Ovarian cancer
OCSCs: Oral cancer stem cells
OPN: Osteopontin
OS: Osteosarcoma
PARP-1: Poly [ADP-ribose] polymerase 1
PC: Prostate cancer
PDE4D: Phosphodiesterases 4D
PI3K: Phosphatidylinositol 3-kinase
piRNAs: Piwi-interacting RNAs
PRC2: Polycomb-repressive complex 2
PSEN1: Presenilin 1
PTC: Papillary thyroid carcinoma
PTEN: Phosphatase and tensin homolog
PTX: Paclitaxel
PUS: Pseudouridine synthases
PVT1: Plasmacytoma variant translocation 1
RA: Rosmarinic acid
RCC: Renal cell carcinoma
RNAi: RNA-mediated interference
RNF2: Ring finger protein 2
RNU2-1f: Fragments derived from U2 snRNA
S100A7: S100 calcium-binding protein A7
SAV1: Salvador homolog 1
SCAI: Suppressor of cancer cell invasion
SCLC: Small cell lung cancer
SIK2: Salt-inducible kinase 2
siRNAs: Small interfering RNAs
Sirt1: Silent information regulator 1
SNORAs: Box H/ACA snoRNAs
snoRNAs: Small nucleolar RNAs
snoRNPs: Small nucleolar ribonucleoproteins
snRNAs: Small nuclear RNAs
snRNPs: Small nuclear ribonucleoproteins
SOX7: Sex determining region Y-box 7
SP1: Specificity protein 1
STAT3: Signal transducer and activator of transcription 3
TAM: Tamoxifen
TCF4: Transcription factor 4
TEAD: TEA domain
THP: Pirarubicin
TMZ: Temozolomide
TNBC: Triple-negative BC
TP53INP1: Tumor protein p53-inducible nuclear protein 1
TUG1: Taurine-upregulated gene 1
TUTases: Terminal Uridylyl Transferases
U: Uridine
UCA1: Urothelial cancer-associated 1
USNORDs: Box C/D snoRNAs
USP22: Ubiquitin-specific peptidase 22
UTR: Untranslated region
Wnt6: Wingless-type MMTV integration site family member 6
XIAP: X-linked inhibitor of apoptosis
ZEB1: Zinc finger E-box binding homeobox 1
ψ: Pseudouridine
